# PReS-FINAL-2112: Mapping the treatment effect of infliximab on a case of refractory anterior uveitis related to juvenile idiopathic arthritis (JIA)

**DOI:** 10.1186/1546-0096-11-S2-P124

**Published:** 2013-12-05

**Authors:** V Miranda, C Zilhão, P Menéres, M Guedes

**Affiliations:** 1 Ophthalmology, Hospital de Santo Antonio, Porto, Portugal; 2 Pediatrics, Hospital de Santo Antonio, Porto, Portugal

## Introduction

JIA related uveitis usually have an insidious onset and chronic evolution. Some remain refractory to treatment with classic immunomodulators and topical steroids, but have been shown to respond to the addition of biological agents (anti-tnfα). When to start biologicals, how to monitor its ocular response and when and how to wean it, remain a matter for debate. The SUN Working Group scales of cell and flare grading remain the most commonly used method of monitoring intra-ocular inflammation but have clear limitations on the follow-up of chronic cases. The measuring of anterior chamber flare with a Flaremeter is more accurate but less frequently used.

## Objectives

To report a case of refractory JIA uveitis treated with infliximab, illustrating the importance of flare measurements in the detection of incomplete response and in infliximab dose optimization.

## Methods

Restrospective review of a case file.

## Results

BMF, female, caucasian, developed arthritis of the right ankle at 3 years of age, followed 3 months later by arthritis of the 3rd, 4th and 5th left hand proximal interphalangeal joints and 4th right foot interphalangeal joint. She was classified as an anti-nuclear antibodies negative, rheumatoid factor negative polyarticular JIA, responding well to non steroidal anti-inflammatories and methotrexate (MTX) 10 mg/m^2^/wk, and achieving complete remission after 6 months of treatment. At the 9th month after diagnosis, bilateral anterior uveitis was diagnosed, thereafter recurring 3-4 times/year over the next 4 years, despite increasing MTX to 15 mg/m^2^/wk. Switching to cyclosporin added unnaceptable side effects with no ocular improvement. At the 5th year after diagnosis (2009), she developed right knee arthritis and was started on etanercept, with articular remission but no ocular improvement. 6 months later, she was switched to adalimumab, with no ocular benefit. After 4 months (2010), she was switched to infliximab 4.4 mg/kg/6/6 weeks, developing uveitis remission. By then, best corrected visual acuity (BCVA) remained 20/50 in each eye due to cataracts, and there were multiple posterior synecheae and band keratopathy. During the next 2 years no anterior chamber cells were detected and flare remained stable at 2+ in each eye, but BCVA gradually declined until 20/200 due to worsening cataracts. A closer follow-up with flaremeter measurements revealed a recurring wave-like pattern of intraocular inflammation with a minimum value of 538 phot/msec on the 2nd week of infliximab and a maximal value on the sixth week of 615 phot/msec. A trial of topical prednisolone acetate managed to further decrease the flare to a minimum of 490 phot/msec on the 2nd week of infliximab, confirming inadequate intra-ocular inflammation control.

## Conclusion

Current biomicroscopic standards of grading intra-ocular inflammation are unsatisfactory for the follow-up of chronic refractory anterior uveitis. In these specific cases, mapping the intraocular inflammation with a more objective tool seems advantageous, revealing variations of intraocular inflammatory status otherwise undetected on biomicroscopy. This may prove useful for both the ophthalmologist and pediatric rheumatologist in refining the optimum therapeutic dose of biological agents.

## Disclosure of interest

None declared.

